# Development and Validation of Age-Friendly Employment Measurement

**DOI:** 10.1093/geroni/igaa057.210

**Published:** 2020-12-16

**Authors:** Mi Sun Choi, Holly Dabelko-Schoeny, Katie White, Marisa Sheldon

**Affiliations:** 1 The Ohio State University, Columbus, Ohio, United States; 2 Age Friendly Columbus, Columbus, Ohio, United States

## Abstract

Scholars have paid attention to the concept of age-friendly to address aging workforce issues. Although prior research has been conducted to conceptualize age-friendly work environments by investigating older workers’ perspectives, little is known about the practices and conditions that promote organizations to employ older employees longer and theory driving measurement of age-friendly employment (AFE). To address these knowledge gaps, we developed the AFE measurement tool based on the existing measures, focus group data, two-round Delphi study and a pilot test. We collected primary data from human resource professionals working in a large city in the midwestern state and evaluated the reliability and validity of the AFE measurement using exploratory factor analysis and confirmatory factor analysis. Results supported the hypothesized five-factor structure. We obtained 16 indicators of AFE: 1) accommodation: fewer physical work demands, reduced work hours, mobility and transportation support; 2) development: career advice, a training needs assessment, and training opportunities to employees all ages; 3) maintenance: financial and medical benefits for full-time workers of all ages, and wellness programs benefits for full-time and part-time workers; 4) utilization: move into a different position, involvement in decision making, knowledge or skills transfer). Accommodation, maintenance, and inclusion factors were predicted by organizational size. A major strength of this study was that the AFE measurement was constructed using a lifespan theory (Selection Optimization Compensation model). The findings of the current study enable employers to self-monitor their ability to employ and retain older employees, especially for small organizations with less than 20 employees.

